# Large language models linking traditional Chinese medicine knowledge and clinical practice

**DOI:** 10.1186/s13020-026-01489-8

**Published:** 2026-10-08

**Authors:** Chenyue Li, Xiaoli Zhang, Longjun Zhu, Bin Zhu, Chu Chu, Xiaodi Li, Yuening Yao, Yuqun Zeng, Jianlan Zheng

**Affiliations:** 1 Urology and Nephrology Center, Department of Nephrology, Zhejiang Provincial People’s Hospital, Affiliated People’s Hospital, Hangzhou Medical College, Hangzhou, 310014 China; 2 Translational Medicine Center, Zhejiang Provincial People’s Hospital, Affiliated People’s Hospital, Hangzhou Medical College, Hangzhou, 310014 China; 3 School of Clinical Medicine, Hangzhou Normal University, Hangzhou, 311121 China; 4 College of Pharmaceutical Science, Zhejiang University of Technology, Hangzhou, 310014 China; 5 Department of Artificial Intelligence and Informatics, Mayo Clinic, Rochester, 55905 USA; 6 Intensive Care Unit, The Affiliated Hospital of Jiangxi University of Traditional Chinese Medicine, Nanchang, 330006 China

**Keywords:** Large language model, Traditional Chinese medicine, Multimodal learning, Knowledge-enhanced models, Clinical decision support

## Abstract

**Background:**

Traditional Chinese medicine (TCM) is a complex medical system characterized by multi-source data, implicit knowledge representation, and syndrome-based diagnostic and therapeutic reasoning. With the increasing digitization of classical texts and clinical records, large language models (LLMs) have attracted growing interest as computational tools for organizing and utilizing knowledge related to TCM. However, existing studies remain scattered, and a comprehensive overview of data resources, modeling strategies, evaluation practices, and application scenarios is still lacking.

**Overview:**

In this review, we summarize current research on the application of LLMs in TCM based on published literature. We focus on commonly used data sources, including classical texts, clinical records, and related structured resources, and review representative modeling approaches such as knowledge-enhanced and multimodal methods. Domain-specific training and fine-tuning strategies, as well as reported evaluation practices, are also summarized. Furthermore, we review representative application scenarios described in the literature, including medical consultation support, syndrome differentiation assistance, prescription-related support, education, and research assistance. Key limitations, such as limited interpretability, safety concerns, and the lack of standardized evaluation frameworks, are discussed.

**Conclusion:**

By organizing existing studies along the workflow from data construction to model development, evaluation, and application, this review aims to clarify the current research landscape and highlight methodological challenges that should be addressed to support cautious and appropriate use of LLMs in TCM research and practice.

**Supplementary Information:**

The online version contains supplementary material available at https://doi.org/10.1186/s13020-026-01489-8.

## Introduction

For millennia, traditional Chinese medicine (TCM) has stood as a cornerstone of healthcare in China and a vital component of global medical traditions. It is a living knowledge system built upon a foundation of holistic philosophy and systemic complexity, linking nuanced observations of symptoms with dynamic interpretations of bodily states to deliver individualized therapeutic strategies [1, 2]. Its core practices, such as syndrome differentiation (*Bian Zheng Lun Zhi*) and the use of multi-component herbal formulas, represent an early and sophisticated form of personalized medicine, making it a crucial element of future medical paradigms [3]. However, the complexity underlying the clinical strength of TCM, including its reliance on context-dependent semantics, layered diagnostic logic, and non-standardized descriptors, has posed a substantial challenge to modern scientific validation and computational modelling [4, 5]. For decades, traditional data mining and statistical approaches have struggled to capture the intricate, hidden relationships that define TCM diagnostics and therapeutics, often resulting in a "black box" critique that has limited its broader integration [6].

The recent evolution of artificial intelligence (AI), particularly the advent of large language models (LLMs), presents a paradigm shift. This moves beyond mere computational tools to offer new cognitive frameworks for understanding TCM's complexity. While early machine learning models showed promise, it is the breakthrough of LLMs, with their ability to process unstructured text, learn abstract relationships, and generate coherent reasoning chains, that offers a path to systemic simulation [7, 8]. These capabilities allow models to align classical texts with contemporary clinical narratives, integrate heterogeneous data and reconstruct the multi-step structure of TCM diagnostic and therapeutic reasoning [9]. Emerging TCM-specific models (TCM-LLMs) have demonstrated the ability to interpret classical descriptions, summarize clinical records and propose preliminary formula adjustments [10], suggesting that computational support for TCM may now be attainable at an unprecedented scale.

This review aims to provide a comprehensive survey of the burgeoning field of LLMs for TCM. We will explore the end-to-end pipeline, from the construction of specialized data corpora and knowledge-enhanced model architectures to the development of domain-specific evaluation benchmarks and the implementation of safe, reliable applications. This work emphasizes the necessity of a closed-loop ecosystem that integrates data, models, evaluation, and safety to unlock the full potential of LLMs in advancing the practice and science of TCM.

## TCM data sources and corpus construction

**Fig. 1:**
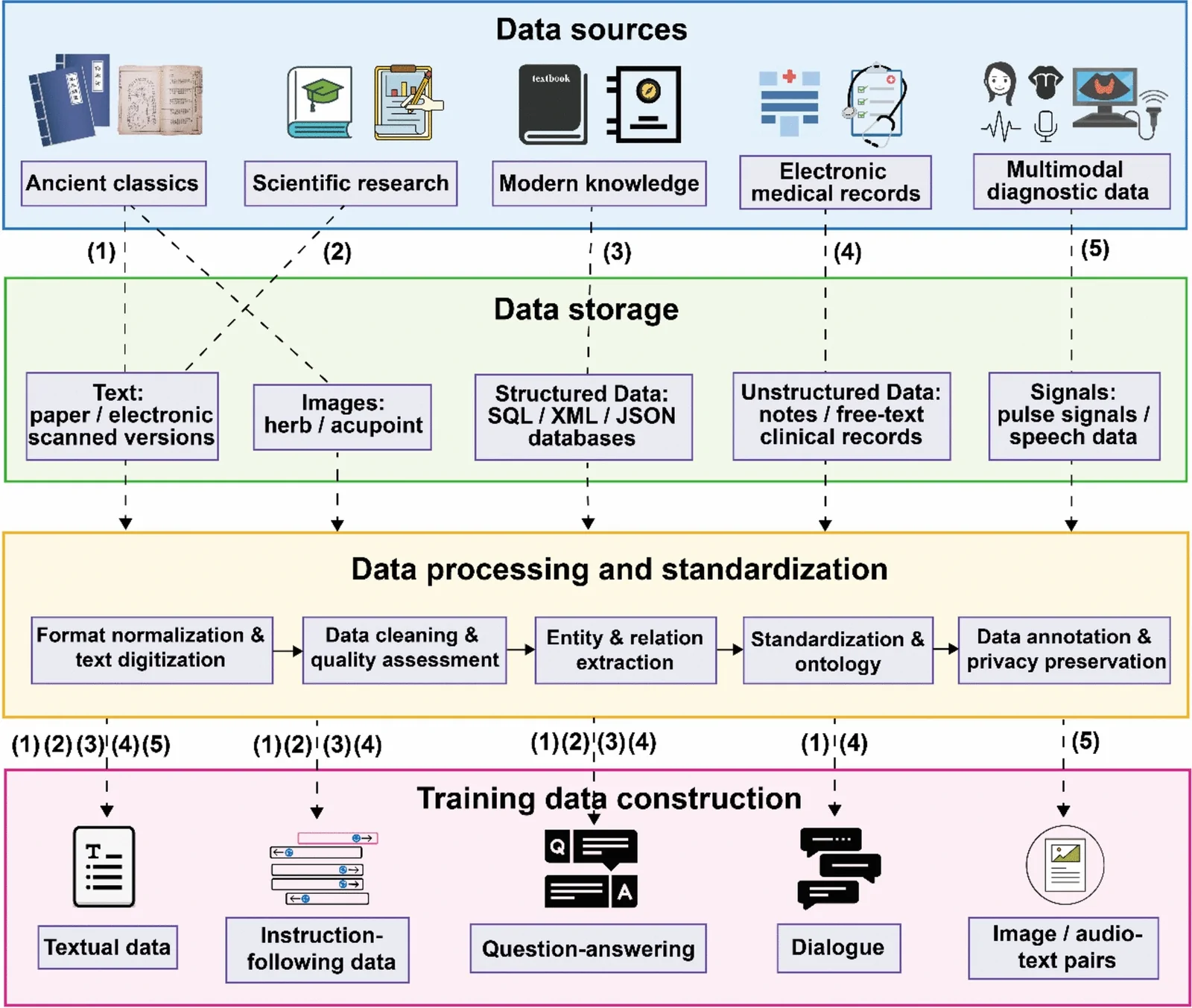
Overview of data sources and types for TCM-LLMs

A high-quality dataset serves as the foundation for constructing TCM-LLMs, determining the upper limits of model performance in knowledge coverage, clinical applicability, and reasoning reliability. This section introduces data types, processing methods, standardization strategies, and the training data framework (Fig. 1).

### Data types and sources

Currently, the data available for TCM are categorized into five primary types: ancient TCM classics, modern TCM knowledge, electronic medical records (EMRs), scientific research, and multimodal diagnostic information. Forging these into a coherent, machine-readable knowledge base suitable for LLMs is the foundational challenge.

#### Ancient TCM classics

Ancient TCM classics documented traditional TCM experience, such as fundamental theories (Yin Yang), diagnostic reasoning processes, syndrome patterns, and herb usage [11, 12]. These contents exist in various forms, including original paper-based manuscripts, woodblock prints, modern digitized versions and electronic scans. They encompass not only textual content but also rich visual data, such as hand-drawn illustrations of medicinal herbs and diagrams of acupuncture points and meridians. Historically, these classics took shape over several millennia and were preserved predominantly in ancient texts written or printed in classical Chinese [13], often characterized by concise syntax, implicit semantics, and strong contextual dependency. With the emergence of comprehensive digital repositories, such as the *Zhonghua Yidian* database containing millions of structurally annotated entries across approximately 5000 classical texts, ancient classics had become a pivotal resource for uncovering evidence-based foundations in TCM [14].

#### Modern TCM knowledge

Modern TCM knowledge serves as standardized, structured, and clinically codified complements to ancient classics. This knowledge is stored in diverse formats, including structured entries in databases (e.g., SQL) [15], digital documents (e.g., PDF, TXT), and web-based knowledge portals. Representative resources include TCM textbooks, national terminological standards, publications of different TCM schools of thoughts, package insert, health-focused forums and integrated databases [16–19]. Presently, large-scale integrated TCM datasets have been developed, presenting diseases, symptoms, syndromes, herbal medicines, formulas, chemical constituents, molecular targets, and herbal images in machine-readable formats to achieve systematic encoding of TCM knowledge [20–23].

#### Electronic medical records

Electronic medical records (EMRs) capture authentic real-world clinical data from routine TCM practice, encompassing individual medical cases (*Yi’an*) and longitudinal patient cohorts. These records typically include basic patient demographics, chief complaints, findings from the four diagnostic methods, syndrome differentiation results, personalized prescriptions, treatment measures, treatment responses, and follow-up outcomes [24, 25]. Driven by the advancement of digital TCM, EMR systems now cover 92% of tertiary TCM hospitals in China, accumulating over 5 billion structured TCM diagnosis and treatment records annually [26]. Importantly, EMRs preserve the sequential clinical reasoning process underlying TCM decision-making, from symptom presentation and syndrome differentiation to prescription formulation and dynamic adjustment over time.

#### Scientific research

Scientific research literature provides continuously updated knowledge that complements classical TCM theory and modern clinical practice. Peer-reviewed studies including mechanistic investigations, pharmacological analyses, randomized controlled trials, and cohort studies, offer critical insights into syndrome pathophysiology, herb-target interactions, and therapeutic efficacy [27, 28]. Large-scale digital repositories, such as the China National Knowledge Infrastructure (CNKI), Wanfang Data, and PubMed, index millions of TCM-related articles and conference proceedings, thereby enabling systematic mining of contemporary TCM scientific finding.

#### Multimodal diagnostic information

Beyond inquiry-based questioning, TCM relies extensively on visual, auditory, and sensory observations, including tongue diagnosis, facial complexion assessment, pulse diagnosis, and auditory cues derived from speech, breathing, and phonation during *Wen* (listening and smelling) diagnosis. Large-scale multimodal repositories have emerged, capturing high-resolution tongue images, facial complexion data, pulse waveforms, phonation signals, and other physiologically relevant modalities [29–31]. These datasets are often paired with symptom annotations, syndrome labels, and treatment outcomes, providing richly correlated cross-modal information for computational modeling.

### Data processing and standardization

Raw TCM data are inherently heterogeneous and largely unstructured, reflecting the long historical evolution, regional diversity, and individualized clinical practices of TCM. Preparing such data for LLM training therefore requires a rigorous, multi-stage pipeline encompassing data format normalization, text digitization, cleaning, semantic structuring, and standardization. This pipeline transforms fragmented raw materials into high-quality, machine-readable corpora suitable for data-driven and knowledge-enhanced LLMs.

#### Data format normalization and text digitization

TCM data originate from diverse sources, including ancient medical classics, modern textbooks, clinical case records, handwritten notes, and scanned documents, resulting in a wide range of formats such as images, PDFs, and heterogeneous text files. As an initial step, format conversion is performed to normalize these materials into unified textual representations. Optical character recognition (OCR) technologies, often customized for classical Chinese and medical terminology, are employed to digitize scanned or handwritten documents. However, due to the presence of rare characters, variant glyphs, and archaic expressions, OCR-generated texts frequently contain errors [32]. Consequently, manual proofreading and expert curation by trained TCM scholars remain indispensable to ensure textual fidelity and semantic accuracy [33]. When conducting cross-language modeling, the selected corpus must be further translated into modern Chinese or English. This process typically combines machine translation with revisions by domain experts to preserve medical semantics and minimize translation-induced interference.

#### Data cleaning and quality assessment

Following digitization, systematic data cleaning procedures are applied to improve corpus quality. These include correcting typographical and OCR-induced errors, normalizing punctuation, terminology, and measurement units, and removing incomplete, irrelevant, or contradictory records. Deduplication is particularly important in TCM corpora, as overlapping content frequently arises from multiple editions of classical texts or repeated clinical case reports [17]. Quality assessment is commonly performed via expert review, with low-quality or ambiguous samples being filtered out to minimize noise propagation and mitigate hallucination risks during LLM training.

#### Entity extraction and categorization

After low-level preprocessing and quality control, the cleaned corpora are subjected to semantic structuring through entity extraction and categorization. Entity extraction represents a foundational task for converting unstructured TCM text into structured knowledge [34]. This involves developing a comprehensive entity system that includes diseases, symptoms, syndromes, formulas, herbs, and functions. Early work relied on models like BERT and ERNIE for named entity recognition (NER) [35, 36]. Modern approaches employ advanced architectures such as BERT-BiLSTM-CRF, which combines bidirectional transformer encoding with recurrent neural networks and conditional random fields to achieve higher accuracy in identifying TCM-specific entities [37]. TCMERE, a framework integrating domain-adaptive models, multi-feature word fusion encoding, and graph convolutional networks, achieves precise extraction of complex entities and their semantic relationships in traditional Chinese medicine, effectively addressing entity nesting and relationship overlap [38].

#### Standardization and ontology construction

Building upon the extracted entities, standardization and ontology construction are performed to resolve semantic ambiguity and establish a unified knowledge framework. Due to the influence of textual errors, regional dialects, and usage habits over thousands of years of transmission, TCM lacks a unified naming system [39]. To address this issue, extracted entities are mapped to standardized terminologies and structured ontologies. Projects like the GFO-TCM and HerbKG provide a structured, hierarchical representation of TCM concepts and their relationships, which is critical for knowledge-driven LLMs [40, 41]. These ontologies reduce ambiguity by assigning canonical names, clarify semantic relationships to support logical reasoning, and promote interoperability across datasets, institutions, and intelligent systems.

#### Data annotation and privacy preservation

To support supervised learning and robust evaluation, high-quality annotated datasets are constructed on top of the standardized corpora. Data annotation typically follows a multi-stage strategy that combines automated labeling with expert annotation by experienced TCM practitioners, followed by iterative human verification. Given the sensitivity of clinical data, privacy-preserving measures are essential [42]. Techniques such as data de-identification, access control, and federated learning frameworks are increasingly adopted to ensure patient privacy while enabling models to learn from real-world clinical data [43, 44]. These safeguards are critical for the ethical and sustainable development of LLMs in TCM applications.

### Training data construction

The performance of any TCM-LLM across different downstream tasks is fundamentally determined by the data it is trained on [45]. The currently available datasets are categorized into several main types: textual data, question-answering (QA), dialogue, medical image/audio-text pairs, and instruction-following data (Table 1). Together, these data types support different stages of model pre-training and fine-tuning.

**Table 1:** Summary of typical medical datasets for pre-training and fine-tuning

Datasets	Type	Data scale	Description	Link
TCMCorpurs[16]	Textual data	3.4 B tokens	Unstructured text collected from the National planned textbooks for higher education in TCM, publications of different TCM schools of thought, and TCM research articles	NA
cMedQA2[47]	QA	100 K questions and 200 K answers	Chinese medical questions and answers collected from an online Chinese medical question answering forum	https://github.com/zhangsheng93/cMedQA2
webMedQA[48]	QA	63 K	Real-world Chinese medical QA collected from online health consultancy websites	https://github.com/hejunqing/ webMedQA
Huatuo-26 M[49]	QA	26 M	Chinese medical QA dataset collected from online medical consultation, knowledge bases and encyclopedias	https://github.com/ FreedomIntelligence/Huatuo-26 M
CMtMedQA[51]	Dialogue	70 K	Multi-round conversation datasets from real doctor-patient conversations	https://github.com/SupritYoung/Zhongjing
BianQue Corpus[50]	Dialogue	2.4 M +	Multi-turn health conversations collected from real-world	https://github.com/scutcyr/BianQue
TCM-Corpus-1B[46]	Dialogue	20 GB	Dataset collected from Baidu Baike, Wikipedia and other sources	NA
TCM Image-Text Data[52]	Image-Text Data	40.6 GB	TCM image-text pairs generated from images and their context using multimodal LLMs	NA
TCM Audio-Text Data[52]	Image-Text Data	75.72 GB	Chinese audio-text pairs in the domain of TCM	NA
CMeKG-8 K	Instruction-following data	8 K	Instruction data based on Chinese medical knowledge graph	NA
ShenNong_TCM_Dataset	Instruction-following data	110 MB	Containing 113 K conversations	https://huggingface.co/datasets/michaelwzhu/ShenNong_TCM_Dataset
med-mix-2 M[54]	Instruction-following data	763 K medical instructions and 1.3 M general instructions	Instruction data based on classical and modern Chinese books, notes from folk doctors	https://huggingface.co/datasets/tyang816/MedChatZH
ChP-TCM dataset[55]	Instruction-following data	6 MB	Instruction data based on the Pharmacopoeia of the People’s Republic of China	https://github.com/QLU-NLP/BianCang/tree/main/ChP-TCM

K: thousand, *M* million, *B* billion, *MB* megabytes, *GB* gigabytes, *QA* question-answering, *NA* not reported

#### Textual data

Textual corpora constitute the foundational domain knowledge of TCM-LLMs and are primarily used during the pretraining phase. These large-scale, unstructured text collections encompass classical medical texts, modern textbooks, clinical guidelines, and scientific research articles, enabling models to acquire fundamental vocabulary, theoretical frameworks, and conceptual systems of TCM. For instance, the TCMCorpurs dataset [46], with 3.4 billion tokens, integrates textbooks and academic literature, providing comprehensive coverage of both traditional doctrines and contemporary research. Such corpora allow models to develop a deep, general understanding of TCM principles prior to task-specific adaptation.

#### Question-answering

QA datasets are crucial for fine-tuning a model's ability to retrieve and present specific factual information accurately. These datasets are commonly constructed from clinical data sources and scientific knowledge repositories, including online health forums, medical encyclopedias, and ancient classics resources. Datasets like cMedQA2 and webMedQA provide real-world question–answer pairs that train the model to handle common medical queries [47, 48]. The large-scale Huatuo-26 M dataset [49], containing 26 million QA pairs aggregated from diverse medical knowledge bases, further strengthens factual recall and response precision, supporting both educational use cases and preliminary clinical consultation scenarios.

#### Dialogue

To support conversational competence, TCM-LLMs require dialogue datasets that capture the interactive nature of clinical communication. Such data are derived from anonymized real-world consultations or carefully designed simulated doctor-patient interactions. Datasets including the BianQue Corpus (over 2.4 million conversations) and CMtMedQA (approximately 70,000 conversations) enable models to learn patient narrative understanding, appropriate inquiry strategies, and empathetic response patterns [50, 51]. These dialogue corpora are particularly important for modeling the inquiry process central to TCM practice, where iterative questioning plays a key role in syndrome differentiation.

#### Image/audio-text pairs

TCM diagnosis is inherently multimodal, integrating information from inspection, auscultation and olfaction, inquiry, and palpation. Consequently, the development of advanced TCM-LLMs increasingly relies on multimodal datasets that associate textual descriptions with images or audio signals. Examples include datasets linking tongue or facial images to diagnostic interpretations, as well as herb images paired with botanical and medicinal descriptions. Models such as ShizhenGPT leverage these multimodal corpora to construct holistic representations that approximate the four diagnostic methods [52]. This capability enables models to, for instance, analyze a tongue image and generate corresponding textual interpretations, representing a critical step toward comprehensive AI-assisted TCM diagnosis.

#### Instruction-following data

Instruction-following data represent the most critical component for transforming a general-purpose pretrained model into a controllable and task-oriented assistant [53]. These datasets consist of explicit instructions paired with desired outputs, teaching models to perform user-defined tasks such as summarizing herb functions, extracting syndrome features, or generating prescription recommendations. Representative datasets include CMeKG-8 K, which is derived from structured knowledge graphs, and the ShenNong_TCM_Dataset, which integrates pharmacological knowledge with instruction templates.

In practice, however, instruction data directly extracted from real-world doctor-patient conversations often exhibit inherent limitations. Clinical responses are typically concise, context-dependent, and stylistically heterogeneous, which reduces their effectiveness for instruction tuning [47]. To address this challenge, self-instruction and LLM-assisted data construction strategies are increasingly adopted. Through controlled prompting, brief clinical answers can be transformed into structured, explanatory responses, while diverse instruction formulations are generated to enhance coverage and robustness. Importantly, this process is commonly constrained by predefined templates, domain ontologies, or knowledge graphs and is followed by expert verification, ensuring medical correctness and alignment with TCM principles.

## Structure of TCM-LLMs

### The overall evolution of TCM-LLMs

The application of advanced NLP in began with early pre-trained language models like BERT and ERNIE [56]. These models were primarily used for foundational tasks such as NER on medical texts and classification of clinical notes, achieving significant improvements over previous methods. The subsequent phase saw the rise of general medical LLMs, such as MedGPT [57], which were trained on broad medical corpora. While not specific to TCM, their powerful reasoning and language capabilities could be transferred to TCM tasks through fine-tuning, demonstrating the potential for generative models in this domain. This has led to the current stage: the development of dedicated TCM-LLMs. To clearly illustrate the development trajectory of TCM-LLMs, this section catalogs existing TCM-LLMs (seen in Table 2), explains different training methods, and concludes with a discussion on evaluating model performance.

**Table 2:** Summary of major LLMs applied to TCM, in terms of their release date, architecture, the number of parameters, model development, application scenarios, modality support, availability status, and resource links

Model	Date	Architecture	#Params	Model development	Application scenarios	Modality support	Availability/model status	Link
BenTsao[58]	2023.04	LLaMa-7B	7B	SFT	Medical conversation	Text	Open weights	https://github.com/SCIR-HI/Huatuo-Llama-Med-Chinese
HuatuoGPT[59]	2023.05	Bloomz-7b1mt	7B	SFT, RLAIF	Medical consultation	Text	Open weights	https://github.com/FreedomIntelligence/HuatuoGPT
ShenNong-TCM-LLM	2023.06	Chinese-LLaMA-7B	7B	SFT	Medical consultation	Text	Open weights	https://github.com/michael-wzhu/ShenNong-TCM-LLM
Zhongjing[51]	2023.08	Ziya-LLaMA-13B-v1	13B	PT, SFT, RLHF	Medical conversation	Text	Open weights	https://github.com/SupritYoung/Zhongjing
BianQue[50]	2023.10	ChatGLM-6B	6.2B	SFT	Health conversation	Text	Open weights	https://github.com/scutcyr/BianQue
TCM-GPT[46]	2023.11	BLOOM-7B	7B	PT, SFT	TCM examination, TCM diagnosis	Text	Closed-source	NA
HuatuoGPT-II[60]	2023.11	Baichuan2	7B, 13B	PT, SFT	Medical conversation, TCM diagnosis, medical examination assistance	Text	Open weights	https://github.com/FreedomIntelligence/HuatuoGPT-II
Huang-Di[61]	2024.01	Ziya-LLaMA-13B-V1	13B	PT, SFT, DPO	Classical TCM knowledge answering, TCM consultation, health preservation	Text	Open weights	https://github.com/Zlasejd/HuangDI
MedChatZH[54]	2024.03	Baichuan-7B	7B	PT, SFT	Medical consultation	Text	Open weights	https://github.com/tyang816/MedChatZH
Lingdan[62]	2024.04	Baichuan2-13B-Base	13B	PT, SFT	Medical consultation, herbal prescription recommendations	Text	Open weights	https://github.com/TCMAI-BJTU/LingdanLLM
Qibo[63]	2024.06	Chinese-LLaMA-7B/13B	7B, 13B	PT, SFT	TCM consultation and syndrome differentiation	Text	Closed-source	NA
Sunsimiao-7B	2024.07	Qwen2-7B	7B	SFT	Medical consultation	Text	Open weights	https://github.com/X-D-Lab/Sunsimiao
BianCang[55]	2024.11	Qwen2/2.5-7B, Qwen2.5-14B	7B, 14B	PT, SFT	TCM syndrome differentiation, TCM disease diagnosis, TCM medical knowledge	Text	Open weights	https://github.com/QLU-NLP/BianCang
TCMChat[64]	2024.11	Baichuan2-7B-Chat	7B	PT, SFT	TCM consultation, entity extraction, medical case diagnosis, herb or formula recommendation	Text	Open weights	https://github.com/ZJUFanLab/TCMChat; https://huggingface.co/ZJUFanLab
TCM-KLLaMA[65]	2025.02	Chinese-LLaMA2-7 B	7B	SFT	Herbal prescription recommendations	Text	Closed-source	NA
Tianyi[16]	2025.05	Baichuan2	7.6B	PT, SFT, KTO	TCM exams, TCM syndrome prediction and treatment, TCM prescription generation, TCM domain QA, TCM syndrome and prescription human evaluation, TCM clinical trial result prediction	Text	Closed-source	NA
DoPI[66]	2025.07	Sunsimiao (Expert) + a smaller LLM (Guidance)	7B	SFT	Medical conversation, TCM diagnoses and treatment	Text, image	Closed-source	NA
AcuGPT[18]	2025.07	Qwen2-7B-Instruct	7B	PT, SFT	Medical conversation	Text	Code-only	https://github.com/suda7788/AcuGPT-Agent
ShizhenGPT[52]	2025.08	Qwen2.5-7B, Qwen2.5-32B	7B, 32B	PT, SFT	TCM multimodal diagnoses	Text, vision, sound, smell, pulse	Open weights	https://github.com/FreedomIntelligence/ShizhenGPT
TianHui[33]	2025.10	DeepSeek-R1-Distill-Qwen-14B	14B	PT, SFT	12 TCM basic scenarios	Text	Open weights	https://github.com/JYfantast/TianHui; https://huggingface.co/JYfantast/TianHui
TCM-DS[67]	2025.11	DeepSeek-LLM-7B	7B	SFT	Edible herbal formulas recommendation	Text	Closed-source	NA
ZhiMingTang[68]	2025.11	DeepSeek-R1	671B	SFT	TCM exams	Text	Closed-source	NA
DualKAG-ShenNong[69]	2025.11	Qwen2.5-7B-chat	7B	PT, SFT	TCM consultation	Text	Closed-source	NA
XuanHuGPT[19]	2025.11	ChatGLM2-6B	6B	SFT	TCM question answering and inference	Text	Closed-source	NA

Column “#Params” shows the number of parameters, *M* million, *B* billion, *PT* pre-training. *SFT* supervised fine-tuning, *RLHF* reinforcement learning from human feedback, *RLAIF* reinforcement learning from AI feedback, *DPO* direct preference optimization, *KTO* Kahneman-Tversky optimization, *NA* not reported

Model availability was assessed from the original publications and official project repositories as of 7 July 2026. “Open weights” indicates that at least one official set of model parameters was publicly downloadable, including full model weights, LoRA adapter weights, or weights for selected model modalities. “Code-only” indicates that code, but not model weights, was available; “Closed-source” indicates that neither model weights nor code was publicly available

### Structural features of TCM-LLMs

#### Enhanced understanding of classical Chinese

A core challenge in TCM modernization is the linguistic gap between ancient texts and modern language [70]. Employing a dual-language pre-training strategy may help to incorporate a massive corpus of classical medical literature alongside modern texts. Models like SiKuBERT have been specifically designed for classical Chinese, incorporating linguistic features unique to ancient texts [71]. This allows the model to develop a nuanced understanding of ancient terminology and theoretical concepts, which is essential for accurately interpreting foundational knowledge.

#### Knowledge-augmented generation

To combat the risk of hallucinations and ground the model's outputs in factual knowledge, TCM-LLMs heavily leverage knowledge enhancement mechanisms. For instance, retrieval-augmented generation (RAG) connects the LLM to vast external knowledge bases (e.g., clinical guidelines, pharmacological databases, classical texts) [55]. When a query is received, the system first retrieves relevant documents or data snippets and then provides them to the LLM as context to generate a more accurate and verifiable response. This approach ensures that the model's outputs are grounded in verified information rather than generated from patterns alone. Going a step further, some models integrate directly with structured knowledge graphs (KGs) [65]. This approach, often termed LLM + KG, combines the generative power of LLMs with the explicit relational reasoning of graph neural networks [72]. This hybrid model allows for more complex, multi-hop reasoning over the structured knowledge base, enhancing the precision of the model's inferences. For example, a model might traverse a given case dataset to predict potential herbs for specific symptoms while capturing the complex associations between symptoms and herbs [73].

#### Generation of TCM reasoning chains

The typical hallmark of TCM diagnosis is its structured reasoning process, often summarized as the chain from “symptoms-syndrome differentiation-pathogenesis analysis-formula and herb selection”. Advanced TCM-LLMs are being trained to explicitly generate this entire reasoning chain [42]. Instead of providing just a final recommendation, the model outputs a step-by-step explanation of its diagnostic logic, making the decision-making process transparent and interpretable for clinicians. This transparency is crucial for building trust and enabling clinicians to understand and potentially override model recommendations.

#### Multimodal capabilities

Recognizing that TCM diagnosis is inherently multimodal, researchers are beginning to incorporate other data modalities. Currently, this primarily involves training models to analyze tongue patterns and facial images for diagnostic purposes or to assess constitutional types, as well as identifying acupoints to assist in acupuncture treatment [29, 74, 75]. Models like ShizhenGPT are pioneering this area by integrating visual understanding with textual knowledge, aiming to replicate the holistic “four diagnostics” approach of TCM practitioner [52]. This multimodal integration is particularly important as it more closely mirrors the actual practice of TCM, where visual and tactile information are as important as verbal communication.

### Training and fine-tuning methods

**Fig. 2:**
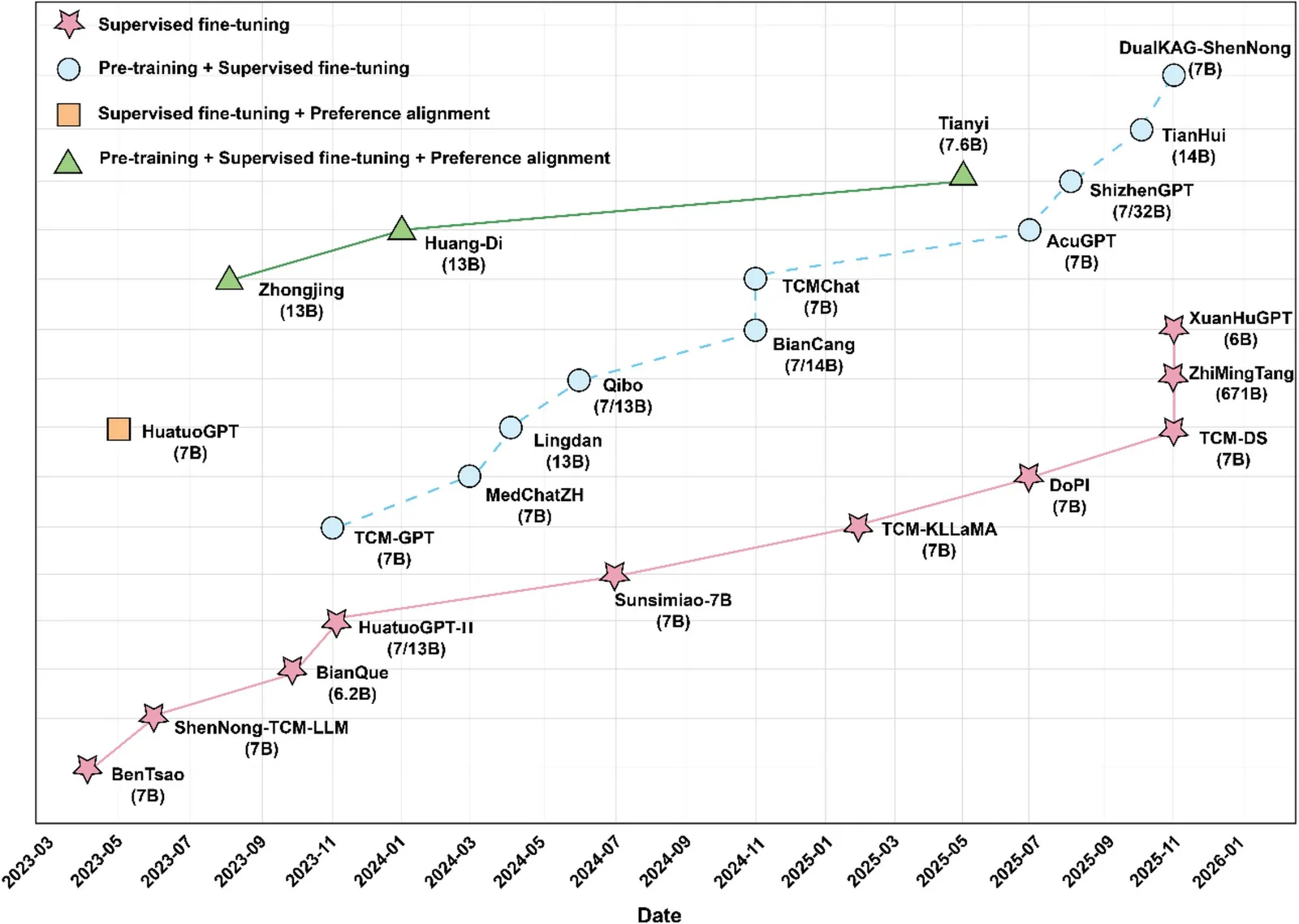
The development of TCM-LLMs over time in different model development types

The training process for a TCM-LLM is a multi-stage endeavor designed to imbue the model with both broad knowledge and specialized skills, progressing from general language understanding to domain-specific expertise. The pre-training corpora and supervised fine-tuning datasets are described in detail in the Data section, while this section focuses on how such data are utilized in different model architectures and training paradigms. Figure 2 demonstrated the development of TCM-LLMs over time in different model development types.

#### Pre-training

PT establishes the foundational language and knowledge capabilities of TCM-LLMs [76]. In this stage, a base language model is trained on a massive mixed-domain corpus that typically includes general web text, scientific literature, biomedical resources, medical textbooks, and a large volume of classical and modern TCM texts. Exposure to such a diverse corpus enables the model to acquire robust general language understanding while simultaneously internalizing TCM-specific vocabulary, theoretical frameworks, and conceptual systems. This joint learning process ensures that the model develops both broad linguistic competence and a foundational understanding of TCM principles, providing a stable basis for downstream adaptation.

#### Supervised fine-tuning

Following pre-training, supervised fine-tuning is performed on carefully curated instruction-response datasets to specialize the model for domain-specific tasks. In the context of TCM, supervised fine-tuning data are designed to cover a wide range of clinically and academically relevant tasks, including medical question answering, syndrome differentiation, herbal formula interpretation, and diagnostic reasoning. Through supervised fine-tuning, the model learns to follow explicit instructions, generate structured and context-aware responses, and generalize to previously unseen task formulations [77, 78]. The quality, diversity, and domain fidelity of the supervised fine-tuning data are critical, as they directly shape the model’s task performance, robustness, and generalization ability.

#### Preference alignment

Preference alignment methods aim to optimize the model’s response distribution according to human or proxy preferences, particularly in complex and safety-sensitive domains such as TCM. Reinforcement learning from human feedback (RLHF) is a widely adopted approach in this stage [79]. Expert practitioners rank or compare multiple model-generated responses to the same prompt, and these preferences are used to train a reward model. The base LLM is then optimized to maximize the reward signal, guiding it toward outputs that are more accurate, helpful, and aligned with clinical reasoning. To reduce annotation costs and improve scalability, reinforcement learning from AI feedback (RLAIF) has been explored [80], where high-quality LLMs or rule-based systems serve as proxy evaluators under carefully designed constraints.

More recently, direct preference optimization (DPO) and Kahneman-Tversky optimization (KTO) have emerged as efficient alternatives that bypass explicit reward modelling [16, 61]. These methods directly optimize the policy using paired preference data, offering improved training stability and reduced computational overhead. Such approaches are particularly appealing for TCM-LLMs, where expert-labeled preference data are scarce and costly, yet nuanced alignment with domain knowledge is essential.

#### Multimodal alignment

For models with multimodal capabilities, an additional training stage is required to align the different data streams. Taking ShizhenGPT as an example, it employs a unified LLM backbone equipped with independent visual and signal encoders to process images and physiological signals [52]. During the critical alignment training phase, supervised learning utilizes image-text and audio-text pairs. An adapter network projects features from each modality into the LLM's shared embedding space, thereby establishing cross-modal semantic mapping and reasoning.

### Benchmark evaluation for TCM-LLMs

Evaluating the capabilities of LLMs in the specialized domain of TCM is a non-trivial task. General-purpose benchmarks are insufficient to assess the nuanced requirements of medical accuracy, diagnostic reasoning, and safety that are paramount in this field. Consequently, researchers have begun to develop dedicated evaluation benchmarks and a multi-dimensional assessment framework specifically for TCM.

#### TCM benchmark datasets

A series of specialized benchmarks have been created to systematically measure the performance of LLMs on various TCM tasks. These datasets are often derived from professional qualification exams, classical texts, and clinical case studies, providing a standardized basis for comparison (Table 3).

**Table 3:** Overview of existing benchmark datasets for LLMs in TCM, in terms of data scale, data source, tasks, format and available links

Dataset	Data scale	Data source	Tasks	Format	Link
Huatuo-26 M[49]	26 M + QA pairs	Online medical QA website, medical encyclopedias, and medical knowledge bases	QA, Dialogue	Text	https://github.com/FreedomIntelligence/Huatuo-26M
TCM-SD[81]	54,152 clinical records	Real-world clinical records	Syndrome differentiation	Text	https://github.com/Borororo/ZY-BERT
TCMD[82]	1500 + questions	High-quality TCM questions from professional practitioners	NER, Term Normalization	Text	NA
TCMBench[83]	5,473 questions	TCM licensing exam	QA	Text	NA
TCM-QA[84]	801 questions	High-quality TCM questions from documents of BaiduWenKu	QA	Text	https://github.com/yizhen-buaa/TCM-QA-datasets
TCM-3CEval[85]	450 questions	Professional knowledge exercise, TCM classics exercise, clinical diagnosis and treatment case	QA	Text	NA
TCMEval-SDT[86]	300 syndrome-diagnosis cases	Internet, classical Chinese medical texts, hospital medical records	Dialogue (syndrome differentiation)	Text	https://github.com/zhuyan166/TCMEval/tree/main/evaluation/TCMEval-SDT
TCM-Eval[68]	6099 questions	National Medical Licensing Examination	QA	Text	https://tcmeval.bamaidical.com
TCM-Ladder[87]	52,000 + multimodal questions	Research, books, exams, online medical QA platforms	MCQ, QA, dialogue, image understanding	Text, images, audio, videos	https://tcmladder.com
MTCMB[88]	7100 (questions, QA pairs, cases)	Real-world case records, national licensing exams, classical texts	TCM knowledge retrieval, clinical reasoning, prescription generation, and safety	Text	https://github.com/Wayyuanyuan/MTCMB
EvalAcu[18]	10,202 questions	TCM university exams, professional certification tests, and curated clinical Q&A	MCQ	Text	NA
TCM-5CEval[89]	2260 questions	Exercise sets from nationally authorized planning textbooks	Clinical research	Text	NA

*M* million, *QA* question and answer, *MCQ* multiple-choice question, *NA* not reported

#### Dimensions of evaluation

**Fig. 3:**
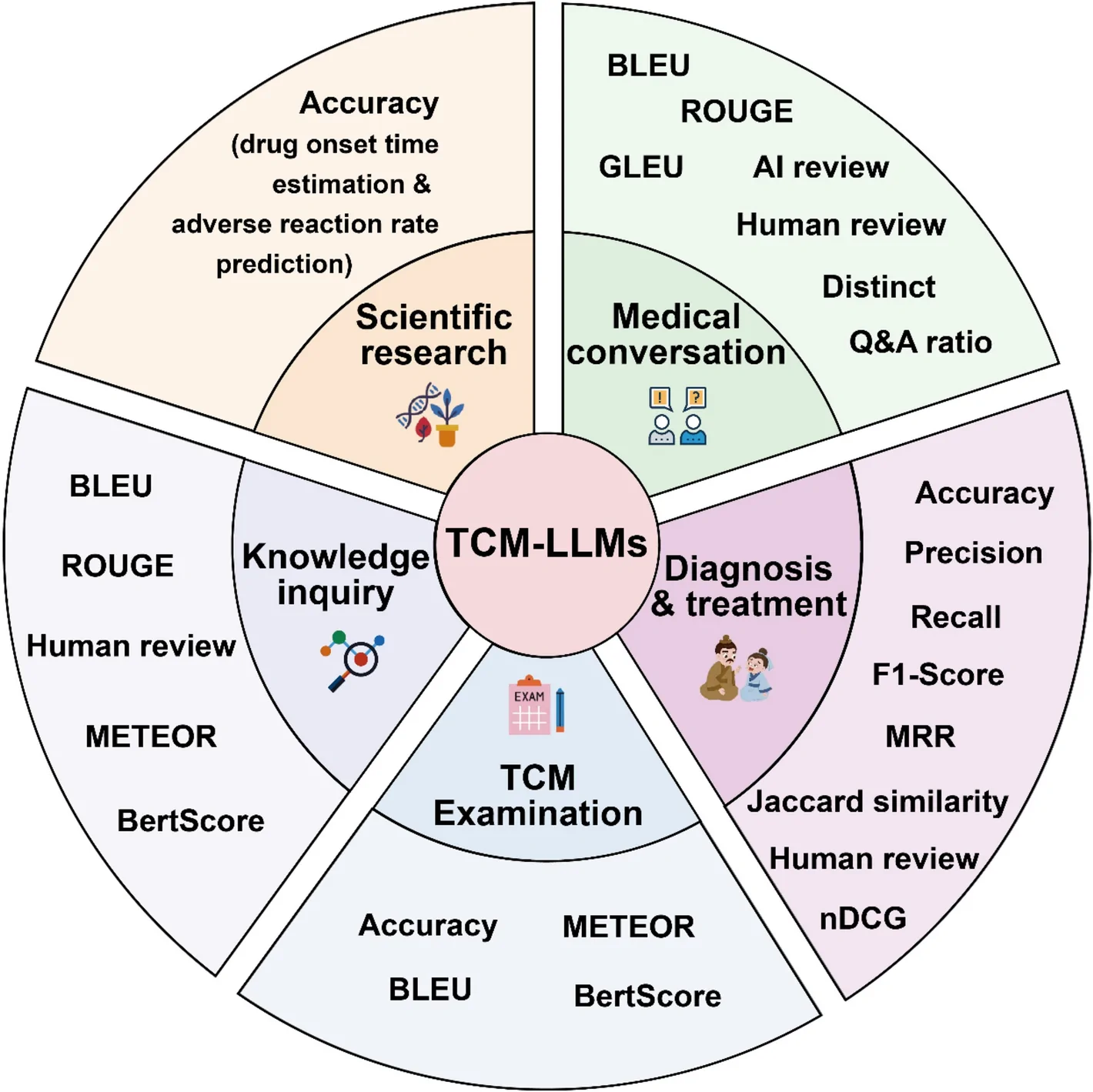
Evaluation metrics for TCM-LLMs across different application scenarios

A comprehensive evaluation of a TCM-LLM must extend beyond simple accuracy scores and encompass a variety of qualitative and quantitative metrics that capture different aspects of TCM model performance (Table 4) in different application scenarios (Fig. 3).

**Table 4:** Comprehensive evaluation metrics used in TCM-LLMs research

Primary dimension	Secondary dimension	Interpretation	Common quantitative methods
Knowledge accuracy Information retrieval ranking	Factual correctness of TCM knowledge	Consistency of disease-syndrome-treatment principle-prescription with classical texts and clinical guidelines	Accuracy/Precision/Recall
	Entity recognition accuracy	Correct identification of diseases, syndromes, herbs, formulas, and acupoints	Precision/Recall/F1-Score
	Knowledge consistency	Agreement between generated content and authoritative TCM knowledge sources	Human expert review/LLM auto-scoring
		Quality of ranked results in knowledge retrieval tasks	MRR/nDCG
Reasoning ability	Completeness of syndrome differentiation logic	Integrity of the reasoning chain during diagnosis	Human expert scoring
	Reasoning consistency	Stability of conclusions across semantically equivalent case descriptions	Human expert review
	Syndrome-prescription matching	Rationality of prescription selection given a specific syndrome	Jaccard similarity/Expert rating
Generation quality	Linguistic fluency	Grammatical correctness and readability of generated text	BLEU/ROUGE/GLUE/Distinct/METEOR
	Semantic coherence	Semantic similarity between generated outputs and reference answers	BERTScore/TCMScore [83]
	Dialogue distinctiveness	Proportion of distinct questions and answers in dialogue	Q&A ratio/Distinct
Clinical usability	Physician agreement	Concordance between model outputs and expert clinician decisions	Human expert agreement
	Decision-support value	Degree to which outputs assist clinical decision-making	Likert scale (e.g., 1–5)
	Retrospective case performance	Performance in retrospective case–control evaluations	Case-level accuracy
Safety assessment	Safety	Accurate, safe, and ethical implications of all the medical entities or phrases mentioned	Human expert review
	Privacy preservation	Avoidance of identifiable personal information leakage	Human expert or AI review
	Humanistic care and respect	Demonstration of empathetic, respectful, and non-stigmatizing communication	Human expert or AI review

Knowledge accuracy: This is the most fundamental dimension. It assesses the factual correctness of the model's outputs. Key metrics include factual consistency (ensuring the generated text does not contradict established TCM principles) and entity correctness rate (verifying the accuracy of identified herbs, acupoints, formulas, etc.). This dimension is critical because even a single factually incorrect recommendation could have serious clinical consequences.

Reasoning ability: This dimension evaluates the model's capacity to emulate the logical processes of a TCM practitioner. This involves assessing the plausibility of the diagnostic reasoning chain (e.g., from symptom to syndrome to treatment principle) and the consistency of formula recommendations with the diagnosed syndrome. The goal is to ensure the model's logic is transparent and clinically sound, not just producing correct answers but doing so for the right reasons.

Generation quality: Generation quality focuses on the linguistic and structural characteristics of the model’s outputs. Key aspects include fluency, coherence, terminological consistency, and the ability to produce well-structured clinical texts such as consultation notes, diagnostic reports, or prescription explanations. High generation quality is essential for clinical usability, as unclear or poorly organized text may obscure critical information and hinder clinician interpretation.

Clinical usability: Clinical usability assesses the practical value of a TCM-LLM in real-world decision-support scenarios. This dimension is commonly evaluated through expert physician ratings, which measure the perceived relevance, helpfulness, and acceptability of model-generated recommendations. Retrospective case–control analyses may also be employed to compare model outputs with real clinical decisions and outcomes [16]. Importantly, such evaluations are conducted in a clinical decision-support context rather than as autonomous medical decision-making.

Safety assessment: Given the high-stakes nature of medical applications, safety assessment constitutes a critical evaluation dimension for TCM-LLMs. This dimension involves quantifying the rate of medically unsafe hallucinations, such as accurate, safe, and ethical implications of all the medical entities or phrases mentioned [51]. In addition to clinical safety, respect for patients and ethical considerations are integral components of safety evaluation [66]. This includes assessing the model’s ability to preserve patient privacy by avoiding the disclosure or reconstruction of identifiable personal information, as well as evaluating its sensitivity to humanistic aspects of care.

## Computing requirements for TCM-LLMs

The development of TCM-LLMs has been closely shaped by the availability of computational resources. Importantly, the purpose of analyzing computing requirements in this review is not to emphasize hardware intensity, but to demonstrate that, relative to general-purpose foundation models, TCM-LLMs typically require substantially more moderate training and inference resources. This characteristic is critical for assessing their feasibility for real-world clinical deployment, particularly in hospital and institutional settings with limited computational budgets (Supplementary Table 1).

Current models exhibit substantial variability in GPU hardware, memory configuration, and training duration, reflecting the diverse research environments across academic institutions and medical centers. Early TCM-LLMs, such as HuatuoGPT and TCM-GPT-7B, were predominantly trained on A100 clusters (40/80 GB)[46, 59], whereas more recent systems, including ShizhenGPT and TianHui, increasingly rely on A800 or H100 GPUs to accommodate larger model sizes, longer contexts, and multimodal inputs [33, 52]. Existing models generally fall into three computational tiers: lightweight systems that train 6-7B parameters on 4–8 GPUs, intermediate models that fine-tune 13B backbones on 8–16 GPUs, and high-end models that require 32 or more GPUs for full-parameter or multi-node distributed training of 14B-32B architectures.

Training time across these models ranges from approximately 12–75 h, depending on model size, optimization strategy, and cluster configuration. While GPU count influences throughput, engineering optimizations, such as Flash Attention 2, gradient checkpointing, and activation recomputation, often play a critical role in reducing memory overhead and accelerating training [90–92]. As a result, competitive performance can be achieved even in moderate compute environments through careful system design.

Given the limited computational budgets of many TCM research groups, nearly all TCM-LLMs adopt memory-efficient training strategies. LoRA and QLoRA (4-bit quantization) enable parameter-efficient fine-tuning of 7B-13B models on as few as four A100 GPUs [51]. DeepSpeed ZeRO technology is also widely used, with ZeRO-2 and ZeRO-3 facilitating optimizer and parameter partitioning that allows full-parameter training under constrained memory conditions [54, 59]. For larger models, such as Qwen2.5-32B-based ShizhenGPT, multi-node distributed parallelism is essential to maintain training stability and throughput [52].

Overall, the computational landscape of TCM-LLMs demonstrates three emerging trends: a gradual transition from A100 to H100/A800 hardware, the universal adoption of parameter-efficient fine-tuning as a standard paradigm, and an increasing reliance on distributed multi-node training for models beyond 14B parameters. These developments indicate that future TCM-LLMs will likely converge toward medium-to-large architectures (14B-30B) supported by optimized training pipelines, balancing computational affordability with the demand for improved clinical reasoning and decision-support capability.

## Applications of LLMs in TCM

**Fig. 4:**
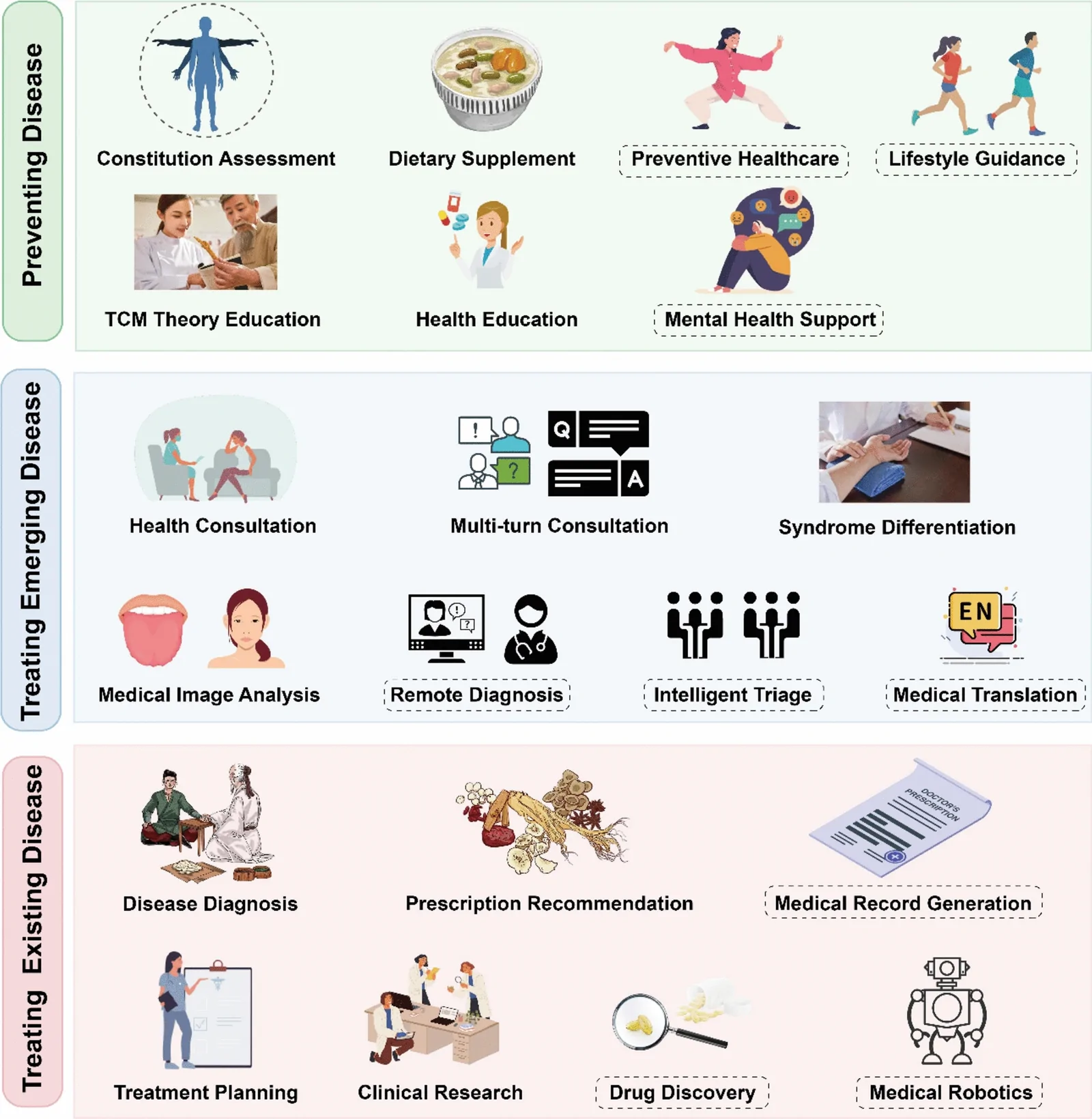
The adaptable applications of TCM-LLMs across the three major clinical scenarios of preventing diseases, treating emerging diseases, and treating existing diseases. The dashed box domains in which mature LLMs have not yet been established

Building upon the advances in data resources, model architectures, and computing infrastructures discussed above, large language models are increasingly transitioning from methodological exploration to practical deployment in Traditional Chinese Medicine. As illustrated in Fig. 4, current applications of TCM-LLMs can be systematically organized along the disease continuum, spanning disease prevention, early-stage intervention, and treatment of established diseases. Across these stages, LLMs support a wide range of tasks, such as health education, medical consultation, syndrome differentiation, prescription recommendation, and research automation.

### Medical consultation

Medical consultation represents one of the most accessible application scenarios of TCM-LLMs, primarily focusing on medical question answering, constitution assessment, and general health consultation. In this setting, models interact directly with users to answer common health-related questions, provide explanations of TCM concepts, and offer personalized constitution-based health advice. Models like HuatuoGPT are designed for interactive dialogue, allowing them to ask clarifying questions to gather more information, mimicking the inquiry part of the four diagnostics [59]. Conversational systems such as Xuanqi Wendui (https://tcmgpt.igancao.cn) are designed to handle initial patient intake, collect symptom descriptions, and deliver educational content in a user-friendly manner. These systems can support long-term health management, subhealth regulation, and preventive care, while serving as a first-line interface that guides users toward appropriate clinical services when necessary.

### Clinical diagnosis and treatment

Clinical diagnosis and treatment constitute the core and most demanding application domain for TCM-LLMs. In this context, models assist clinicians in disease diagnosis, syndrome differentiation, treatment planning, and recommendations of herbal formulas or acupoints. Based on the identified syndrome differentiation, LLMs can recommend classical herbal formulas or even generate novel combinations of herbs [65]. These recommendations are drawn from a vast knowledge base of ancient texts, clinical records, and modern pharmacological data, ensuring they are grounded in both traditional theory and evidence [93, 94]. Beyond conventional medicinal prescriptions, LLMs also show growing potential in supporting edible herbal formulas [67]. Such recommendations are particularly relevant for chronic conditions, subhealth states, and post-treatment recovery, where long-term safety, patient adherence, and preventive regulation are emphasized. A key skill of an experienced TCM practitioner is the ability to modify classical formulas to suit the specific needs of an individual patient. LLMs are also being trained to provide intelligent suggestions for adding or removing herbs based on the patient's specific symptoms, constitution, and comorbidities.

### TCM examinations and professional assessment

TCM-LLMs are increasingly applied in educational and assessment scenarios, particularly for TCM examinations. These applications include automated handling of multiple-choice questions, multiple-answer questions, and reading comprehension tasks commonly used in TCM education and certification exams [33]. By simulating examination conditions, LLMs can support students in self-assessment, provide detailed explanations for correct and incorrect answers, and generate new practice questions aligned with curricular standards. In professional training contexts, such systems can also create simulated clinical cases, enabling students and junior practitioners to practice diagnostic reasoning and treatment decision-making in a controlled environment.

### Knowledge-oriented question answering

Knowledge-oriented question answering focuses on structured access to both classical and modern TCM knowledge. This includes answering questions related to ancient medical texts, interpreting classical terminology, and explaining the pharmacological effects, active components, and mechanisms of herbs. LLMs with strong classical Chinese comprehension can translate and annotate ancient texts, lowering barriers to accessing historical knowledge [95]. They also enable semantic search across large corpora, allowing users to retrieve information based on concepts rather than keywords. Additionally, integration with modern pharmacological and chemical databases allows models to respond to queries about herb constituents, molecular targets, and potential mechanisms, bridging traditional knowledge with contemporary biomedical research.

### Scientific research assistance

In the research domain, TCM-LLMs are emerging as powerful tools for scientific discovery and analysis. They can assist with literature retrieval, automated summarization, hypothesis generation, and knowledge graph construction by integrating information from large-scale classical texts, modern biomedical literature, and clinical datasets [96, 97]. Beyond conventional literature review, recent applications emphasize abstract-driven topic generation and topic-guided abstract writing. More advanced applications include supporting predictive tasks in both retrospective and prospective studies, such as outcome prediction, pattern identification across patient cohorts, and exploratory analysis of treatment effectiveness [16]. By automating time-consuming analytical processes and highlighting potential associations, TCM-LLMs have the potential to accelerate research workflows and support result predication in TCM.

## Future directions and challenges

**Fig. 5:**
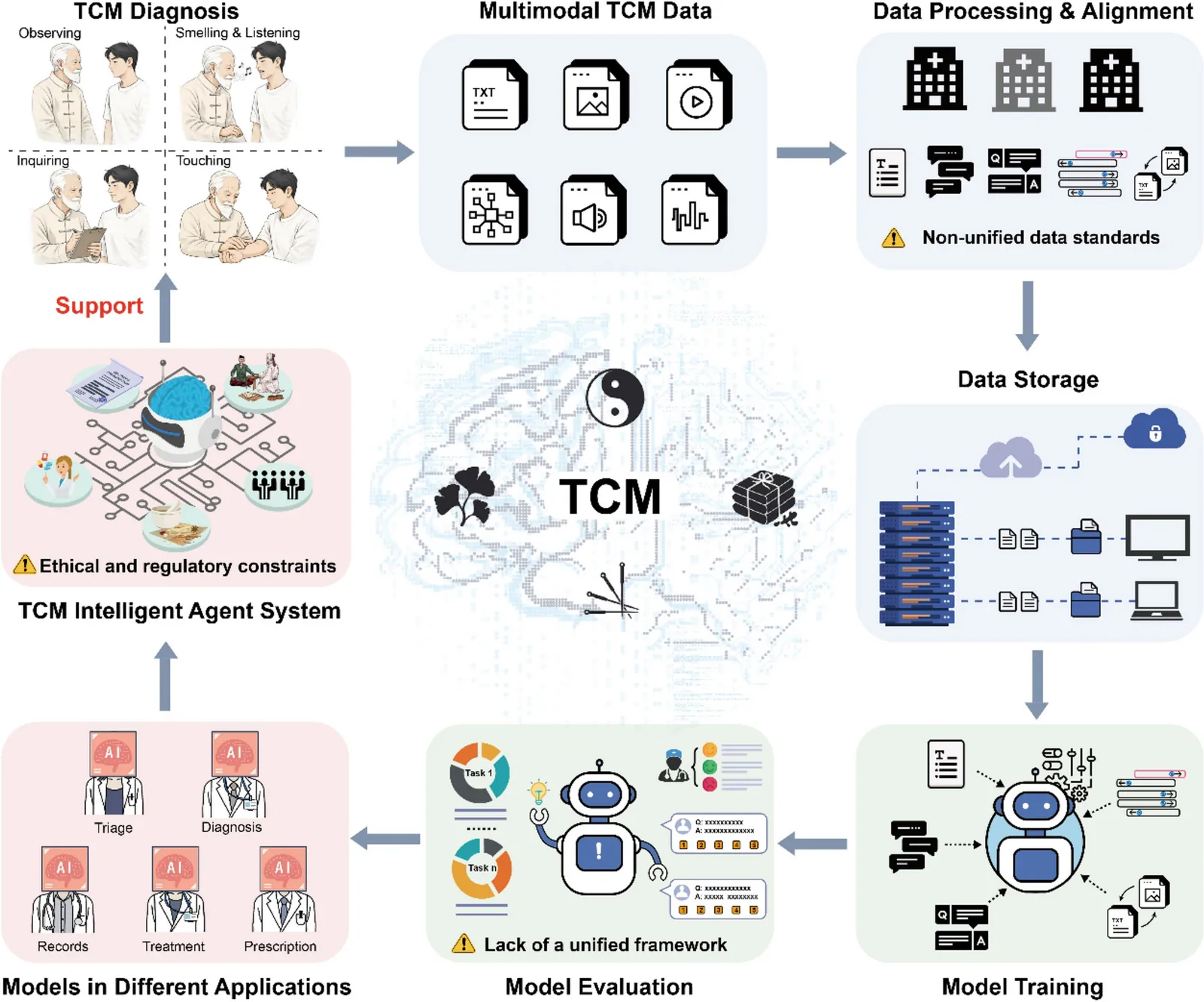
A closed-loop intelligent agent framework highlighting key challenges across the TCM-LLM pipeline

Despite rapid progress, the development of TCM-LLMs remains constrained by challenges that span the entire diagnostic and deployment pipeline. As summarized in Fig. 5, future directions and challenges of TCM-LLMs are best understood as a closed-loop, agent-centered system, linking multimodal TCM diagnosis, data processing and storage, model training and evaluation, and real-world clinical deployment.

### Building a collaborative and standardized data ecosystem

TCM diagnosis fundamentally relies on the integration of inquiry, inspection, auscultation-olfaction, and palpation. Although large-scale datasets of tongue images, facial complexion, pulse waveforms, and audio signals are increasingly available [98–100], the absence of unified data acquisition and annotation standards remains a major barrier to clinical utility [101]. The development and adoption of common data models and annotation guidelines spanning classical texts, electronic health records, and multimodal diagnostic data are essential prerequisites for building the next generation of large-scale, pre-trained models. Such standards would enable researchers from different institutions to collaborate more effectively and would facilitate the creation of larger, more diverse training datasets [102].

However, data-level standardization alone is insufficient to ensure the clinical reliability of multimodal TCM systems. Pulse waveforms are sensitive to sensor architecture, applied pressure, sampling settings, device calibration, and operator procedures [103–105], whereas olfactory information relies on electronic-nose systems whose outputs may vary with sampling protocols, ambient conditions, sensor drift, and device- and site-specific signal distributions [106–108]. These sources of variability may compromise reproducibility and cross-institutional generalizability. Therefore, although multimodal LLM software is advancing rapidly, its routine clinical deployment remains substantially constrained by the limited availability of standardized and clinically validated acquisition hardware, as well as device-level quality-control procedures. Future development should integrate model construction with calibrated sensing devices, unified acquisition protocols, metadata specifications, cross-device calibration methods, and prospective multicenter validation.

### Towards interpretable and theory-aware TCM-LLMs

The critical area of research is enhancing the explainability of the entire clinical reasoning process [109]. Future models must not only generate the “disease-symptom-syndrome-formula-herb-effect” chain but also be able to justify each step with evidence from classical texts, clinical data, and modern research [110]. Frameworks like the DIKWP (Data, Information, Knowledge, Wisdom, Purpose) model are being explored to provide a more structured and semantically transparent representation of this reasoning process [111, 112]. At the same time, the absence of unified and clinically grounded evaluation standards further constrains progress in this area. Existing benchmarks typically assess isolated tasks, such as question answering or syndrome classification, and fail to systematically evaluate reasoning transparency, theoretical consistency, and safety across the full diagnostic workflow. Addressing these limitations requires theory-aware modeling approaches that integrate structured TCM knowledge into both model design and evaluation, alongside standardized, process-level assessment frameworks that support interpretability, comparability, and expert-in-the-loop validation.

### Agent-based systems, safety, and regulation

Increasingly, TCM-LLMs are expected to operate not as standalone chatbots but as components of intelligent, agent-based systems capable of task decomposition, multi-step reasoning, and coordinated decision support. Such intelligent agents can integrate symptom collection, diagnostic reasoning, treatment suggestion, and safety checks within a human-in-the-loop framework, better reflecting real clinical workflows. At the same time, ethical and regulatory considerations become paramount [113]. Regulatory bodies, developers, and clinical institutions must collaborate to establish clear guidelines for the development, validation, and deployment of medical LLM in consultation with TCM experts to ensure they are appropriate for the domain. This includes defining the scope of practice for LLMs, clarifying liability, and ensuring that models are subject to rigorous pre- and post-market surveillance [114]. Recent regional regulations in China explicitly prohibit AI systems from independently generating medical prescriptions without physician oversight. Meanwhile, techniques like RAG have improved factual grounding, but the risk of model hallucination remains [115]. Future work must focus on developing more advanced mechanisms for detecting and mitigating hallucinations. Furthermore, every piece of information or recommendation generated by the model must be traceable back to its source, whether it's a specific passage in a classical text, a clinical trial result, or an entry in a knowledge graph.

## Conclusion

The convergence of LLMs and TCM marks a pivotal moment in the history of this ancient medicinal system. We are witnessing the emergence of a new generation of AI-powered tools that possess an unprecedented ability to understand, process, and generate insights from the vast and complex body of TCM knowledge. From building comprehensive data foundations and designing knowledge-augmented architectures to establishing rigorous evaluation benchmarks and deploying practical clinical applications, the field is advancing at a remarkable pace. These models hold the potential to dramatically enhance diagnostic accuracy, personalize treatment, accelerate research, and make the wisdom of TCM more accessible than ever before.

However, the path forward is fraught with challenges. The inherent complexity and nuance of TCM theory, the critical need for high-quality data, and the profound ethical responsibilities associated with medical AI demand a measured and thoughtful approach. The key to success will lie in fostering a closed-loop ecosystem where data, models, evaluation, and safety are seamlessly integrated, and where deep collaboration between AI experts and TCM practitioners is the norm. By embracing the principles of trustworthiness, safety, and a deep respect for the medical tradition, LLMs are poised not to replace the human practitioner, but to become an indispensable partner in the quest to unlock the full potential of TCM.

## Supplementary Information

Supplementary Material 1

## Data Availability

The data that support the findings of this study are available from the corresponding author upon reasonable request.

## Abbreviations

AI: Artificial intelligence
CNKI: The China National Knowledge Infrastructure
DPO: Direct preference optimization
EMRs: Electronic medical records
KGs: Knowledge graphs
KTO: Kahneman-Tversky optimization
LLMs: Large language models
MCQ: Multiple-choice question
NER: Named entity recognition
OCR: Optical character recognition
PT: Pre-training
QA: Question-answering
RAG: Retrieval-augmented generation
RLAIF: Reinforcement learning from AI feedback
RLHF: Reinforcement learning from human feedback
SFT: Supervised fine-tuning
TCM: Traditional Chinese medicine
TCM-LLMs: TCM-specific LLMs
